# Characterization of the complete mitochondrial genome of *Diplocheila zealandica* Redtenbacher (Insecta: Coleoptera: Carabidae) from Henan province

**DOI:** 10.1080/23802359.2020.1731348

**Published:** 2020-02-28

**Authors:** Bing Fang

**Affiliations:** College of Mathematics and Information Science, Guiyang University, Guiyang, China

**Keywords:** *Diplocheila zealandica*, Carabidae, mitochondrial genome

## Abstract

*Diplocheila zealandica* Redtenbacher is a natural enemy of aphids and larva of lepidoptera worldwide. Here, we first report the characterization of mitochondrial genome of *D. zealandica* in *Diplocheila* and its phylogenetic position. The complete mitogenome (GenBank accession number: MN995217) of *D. zealandica* from Henan Province consisted of a circular DNA molecule of 16,190 bp (with 21.66% G + C content), which comprised 13 protein-coding genes (PCGs), and 22 tRNA, and two rRNA genes. PCGs had typical ATN (Met) initiation codons and were terminated by typical TAN stop codons.

*Diplocheila zealandica* Redtenbacher is a natural enemy of aphids and larva of lepidoptera worldwide. Here, we first report the characterization and phylogenetic studies of the complete mitogenome of *D. zealandica* in *Diplocheila*.

Samples of adult *D. zealandica* (GYU-20190903-001) were obtained from Jingziguan Town (E 111.026°, N 33.244°), Xichuan County, Nanyang City, Henan Province, China on 3 September 2019. Genomic DNA was isolated and fragmented to build a genomic library of the approximate insert size 350 bp that was sequenced (paired end 2 × 150 bp) using an Illumina HiSeq 4000 (Illumina, Inc., San Diego, CA, USA). We obtained 65,749,062 reads of raw data and 65,630,762 reads of high-quality, clean data (99.82%) cleaned by cutadapt version 1.9.1 (https://github.com/marcelm/cutadapt/) (Martin 2011). The mitochondrial genome was assembled *de novo* with Velvet version 1.2.10 (https://github.com/dzerbino/velvet/) (Zerbino and Birney 2008), gapfilled with SSPACE version 3.0 (Boetzer et al. 2011) and GapFiller version 1.1 (Boetzer and Pirovano 2012).

The mitogenome of *D. zealandica* consists of a 16,190 bp circular DNA molecule (GenBank accession number: MN995217), with 40.49% A, 37.85% T, 12.36% C, and 9.30% G, which has an A/T bias (21.66% C + G content). The AT- and GC-skews of the major strands of the mitogenome were calculated to be approximately 0.0337 and 0.1415, respectively. The length of the A/T-rich region in the mitogenome is 1342 bp, with 84.34% A + T content, and is located between the srRNA and tRNA-Ile.

The order and orientation of the functional areas of the *D. zealandica* mitogenome are identical to those in the *Tenebrio obscurus*, *Zophobas atratus*, and *Rhyzopertha dominica* mitogenome (Bai et al. 2018, 2019; Ouyang et al. 2019). The mitogenome of *D. zealandica* contained 13 protein-coding genes (PCGs), and 22 tRNA, and two rRNA genes. All 13 PCGs had typical ATN (Met) start codons and TAN stop codons; *atp8* and *nad1* had ATA as a start codon, *nad3, nad5*, *nad4l*, *nad6*, *nad2*, and *cox1* had ATT as a start codon, and *cox2, atp6*, *cox3*, *nad4*, and *cob* had ATG as a start codon. From these, *atp8*, *atp6*, *cox3*, *nad4*, *nad6, cob*, and *nad2* had a TAA stop codon, *nad1* had a TAG stop codon, *cox1*, *cox2*, *nad3*, *nad4l*, and *nad5* had an incomplete stop codon consisting of a T−, which was completed by the addition of 3’A nucleotides to the resulting mRNA. The 22 tRNA genes were interspersed throughout the coding region and ranged from 65 (trnC, trnI, trnT, trnN, trnR, and trnA) to 72 bp (trnV) in length. lrRNA and srRNA were 1278 and 788 bp long, respectively.

To validate the phylogenetic position of *D. zealandica*, the mitogenome DNA sequences from 11 species of Carabidae were used to construct a phylogenetic tree by the Maximum-Likelihood method using the MEGA 7 software (Kumar et al. 2016) (Figure 1). In conclusion, our study provides information of the mitogenome of *D. zealandica*, which will be useful for molecular identification and phylogenetic studies.

**Figure 1.**
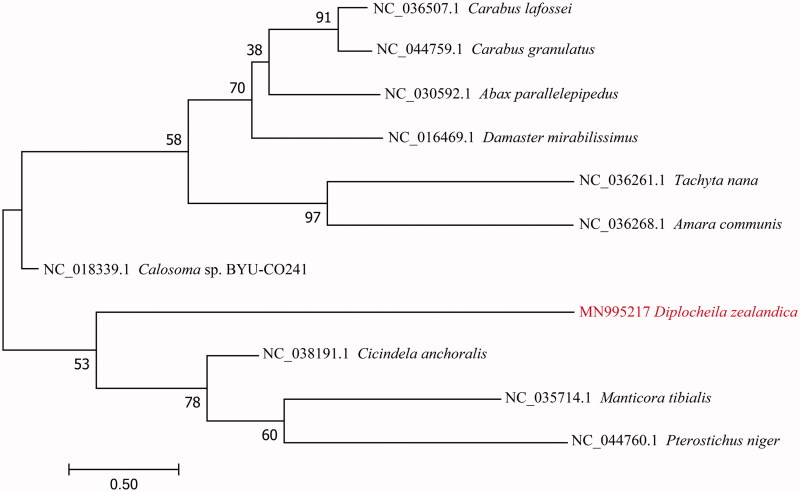
The Maximum-Likelihood phylogenetic tree of *D. zealandica* and other 10 beetles of Carabidae based on the DNA sequences of mitogenome.
